# Isokinetic knee strength as a predictor of cardiorespiratory responses during loaded aerobic capacity test in elite athletes

**DOI:** 10.1038/s41598-025-11429-5

**Published:** 2025-07-17

**Authors:** Bekir Çar, Ahmet Kurtoğlu, Özdemir Atar, Musa Türkmen, Özgür Eken, Mehmet Soyler, Monira I. Aldhahi

**Affiliations:** 1 Department of Physical Education and Sports Teaching, Faculty of Sport Science, Bandırma Onyedi Eylul University, Balikesir, 10200 Turkey; 2 Department of Coaching Education, Faculty of Sport Science, Bandırma Onyedi Eylul University, Balikesir, 10200 Turkey; 3 Department of Coaching Education, Faculty of Sports Sciences, Çanakkale Onsekiz Mart University, Çanakkale, 17000 Turkey; 4 Department of Physical Education and Sports, Institute of Health Sciences, Inonu University, Malatya, 44000 Turkey; 5 Department of Physical Education and Sport Teaching, Faculty of Sports Sciences, Inonu University, Malatya, 44000 Turkey; 6 Social Sciences Vocational High School, Çankırı Karatekin University, Çankırı, 18100 Turkey; 7 Department of Rehabilitation Sciences, College of Health and Rehabilitation Sciences, Princess Nourah bint Abdulrahman University, P.O. Box 84428, Riyadh, 11671 Saudi Arabia

**Keywords:** Knee isokinetic strength, ACT, Cardiorespiratory fitness, Muscle performance, loaded exercise, Physiology, Cardiology

## Abstract

This study aimed to investigate the predictive capacity of knee isokinetic strength parameters on cardiorespiratory responses during aerobic capacity test (ACT). It provides novel insights into the interplay between muscular strength and cardiorespiratory function through comparative analyses of loaded and unloaded ACT protocols in elite athletes. Thirty elite modern pentathlon athletes (age: 21.43 ± 0.77 years) underwent isokinetic knee strength assessments at angular velocities of 60°/s and 240°/s. Cardiorespiratory parameters—average breath volume (ABV), average breath frequency (ABF), auxiliary oxygen density (AOD), and heart rate (HR)—were recorded during the Bruce Protocol conducted under two conditions: unloaded and with a 10 kg loaded vest. Relationships between isokinetic strength metrics and cardiorespiratory parameters were analyzed using linear regression models. There were no significant differences in ABV, ABF, or AOD between loaded and unloaded ACT conditions (*p* > .05). However, HR was significantly lower during loaded ACT (*p* < .05). Linear regression revealed that at 60°/s, several knee strength parameters, including peak torque extension (PT-EXT), peak torque flexion (PT-FLX), total work flexion (TW-FLX), average power flexion (AP-FLX), and agonist strength (AGANT), significantly predicted ABV during loaded ACT (R^2^ = 0.804, *p* = .004). A similar pattern was observed at 240°/s, where comparable predictors explained a significant variance in ABV (R^2^ = 0.761, *p* = .012). No significant predictive relationships were identified during unloaded ACT. Isokinetic knee strength parameters significantly predict cardiorespiratory responses during loaded ACT but not during unloaded protocols. These findings suggest that isokinetic strength assessments may be a valuable tool for optimizing ACT prescription and monitoring training adaptations in elite athletes.

## Introduction

Regular physical activities result in far-reaching benefits to general health, a higher quality of life for athletes, and an increase in critical performances^1–4^. Among various training protocols, High-Intensity Interval Training (HIIT) has garnered widespread acclaim within the field of sports science^3,4^. HIIT typically involves alternating periods of intense effort and rest to achieve physiological gains, but the Aerobic Capacity Test (ACT) is a graded exercise test designed to assess cardiovascular and aerobic capacity and may also serve as a predictor of performance in HIIT training. Additionally, ACT has been comprehensively validated for its effectiveness in improving cardiovascular capacity and metabolic function, making it a preferred method for optimizing fitness outcomes^5^. This methodology demonstrates significant efficacy in enhancing both aerobic and anaerobic capacities, yielding substantial improvements in cardiorespiratory fitness, muscular strength, and metabolic health parameters^6,7^. Current empirical evidence indicates that ACT’s beneficial effects extend beyond cardiovascular health improvements, encompassing significant enhancements in muscular performance and strength parameters^8^.

The physiological adaptations and performance outcomes elicited by ACT protocols demonstrate considerable heterogeneity based on the specific exercise modalities employed and equipment parameters utilized. Empirical investigations have established distinct adaptive responses in muscular strength, endurance capacity, movement amplitude, and velocity metrics between resistance-based and bodyweight exercise interventions^9–11^. While resistance-loaded exercises induce superior mechanical tension, resulting in augmented strength and endurance adaptations, bodyweight exercises facilitate enhanced movement amplitude and velocity parameters through reduced external load constraints^11^.

Isokinetic force parameters are recognized as significant quantitative indicators of muscular force-generating capacity during ACT protocols, demonstrating particular significance in the optimization of strength and endurance adaptations specific to knee joint musculature^12^. Isokinetic dynamometers facilitate the precise measurement of velocity-specific maximum force production capabilities, demonstrating extensive utility in both athletic performance assessment protocols and rehabilitation interventions^13,14^. Knee joint isokinetic strength evaluation protocols constitute essential diagnostic measures for detecting lower limb strength asymmetries in athletic populations. Bilateral strength imbalances between right (R) and left (L) knee articulations may manifest in compromised performance parameters and increased injury susceptibility^15^.

Lower extremity muscle strength, particularly in the knee area, can significantly influence respiratory (ventilatory) and circulatory (hemodynamic) responses during ACT. Increased muscle power production leads to higher oxygen consumption and greater stress on the cardiovascular system. Current literature indicates that individuals with higher muscle strength utilize oxygen more efficiently during exercise and exhibit more balanced cardiorespiratory responses.

Nevertheless, there exists a paucity of research investigating the differential effects of resistance-loaded and bodyweight ACT protocols on knee joint loading patterns and subsequent isokinetic force performance parameters^16^. The present study investigates the interrelationship between bilateral knee isokinetic force production (right [R] and left [L]) and cardiorespiratory parameters during resistance-loaded versus bodyweight ACT interventions. The investigation specifically examines ACT-induced adaptations in cardiorespiratory parameters—including tidal volume, respiratory frequency, and oxygen consumption—while elucidating the associations between knee force capabilities and cardiorespiratory system dynamics. We hypothesized that bilateral knee isokinetic strength parameters would demonstrate significant predictive relationships with cardiorespiratory responses during loaded ACT, but not during unloaded protocols. The resultant findings may inform the development of evidence-based training protocols for optimizing athletic performance outcomes.

## Materials and methods

### Research design and participants

The study cohort consisted of thirty elite male modern pentathlon athletes, aged between 20 and 23 years, with a mean age of 21.43 ± 0.77 years. To determine the appropriate sample size, researchers utilized G-Power 3.1 software (University of Düsseldorf, Düsseldorf, Germany). An a priori power analysis was conducted, setting the effect size (d) at 0.44, the significance level (α) at 0.05, and statistical power (1-β) at 0.80. This analysis indicated a minimum of 28 participants was required, ensuring an actual power of 0.81. The final sample exceeded this threshold, enhancing the study’s robustness. Inclusion criteria mandated a minimum five-year history of competitive participation as licensed athletes, documented participation in national and international championships, and current national team membership status. Participants were excluded from the study if they exhibited any pre-existing chronic medical conditions or respiratory disorders, such as asthma or dyspnea. Additional exclusion criteria included cardiac-related hospitalizations within the past 12 months, active infections, or recent antimicrobial therapy within 30 days prior to study initiation. Moreover, individuals with a history of knee joint pathology—including meniscal injuries, anterior or posterior cruciate ligament damage, or medial and lateral collateral ligament injuries—were also deemed ineligible. These stringent criteria ensured a homogenous and medically stable cohort.

This investigation adhered to the ethical principles outlined in the Declaration of Helsinki and was approved by the Non-Interventional Health Sciences Ethics Committee of Bandırma Onyedi Eylül University (Protocol Number: 2024/123; Approval Date: 17.05.2024). Before enrollment, participants received a detailed briefing from the principal investigator, which covered the experimental procedures, research hypotheses, study objectives, and anticipated contributions to the scientific community. Following this comprehensive explanation, written informed consent was obtained from each participant. The discussion emphasized the voluntary nature of participation, the potential risks involved, and the right to withdraw from the study at any time without penalty. These measures ensured both ethical rigor and participant autonomy.

### Study design

**Fig. 1:**
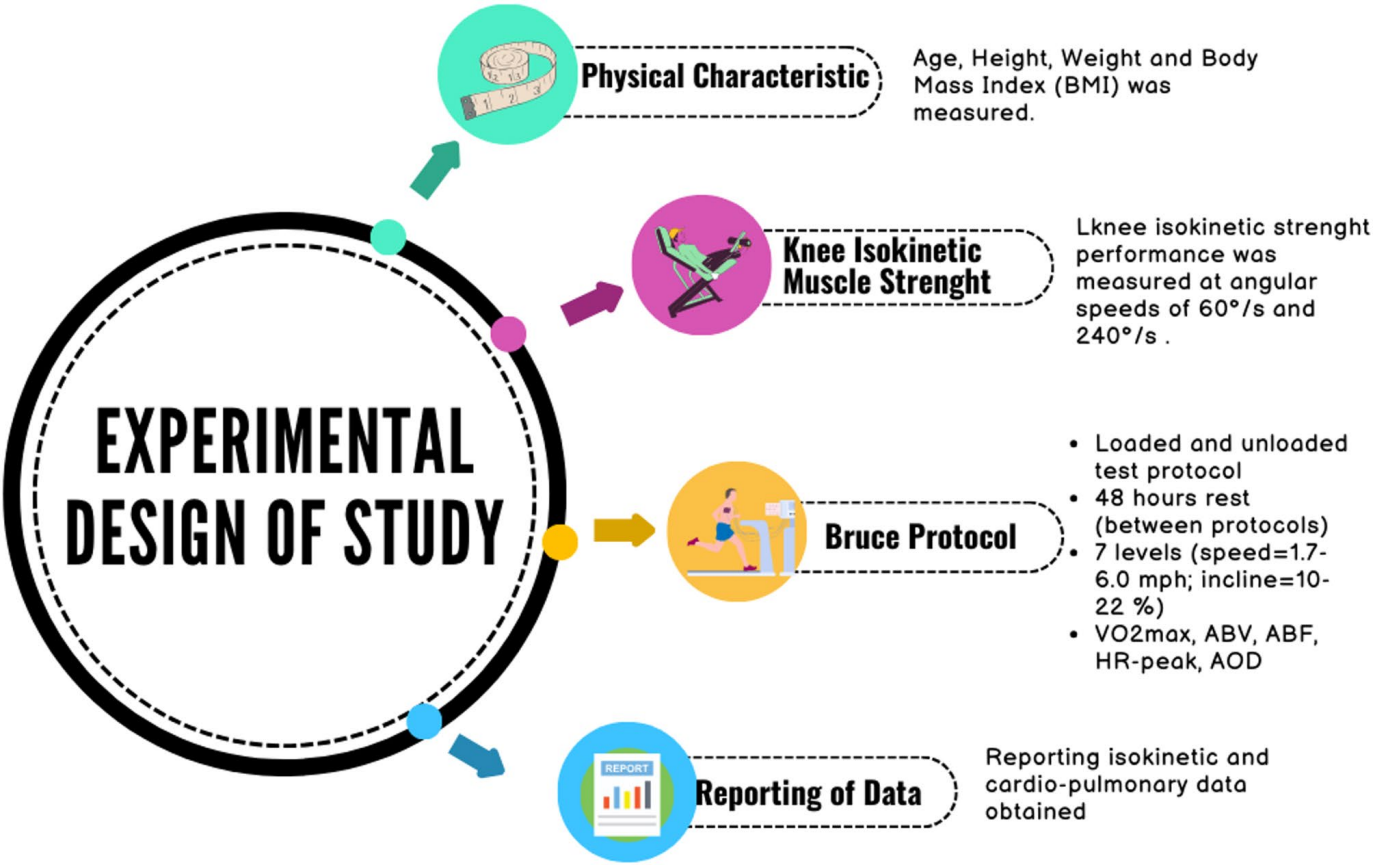
Study design.

The experimental design employed a randomized, single-blind protocol with a singular testing sequence. Anthropometric and demographic parameters, including age, stature, body mass, body mass index (BMI), and body composition analyses, were initially documented. Bilateral isokinetic muscle strength was assessed at two distinct angular velocities: 60°/s and 240°/s. After a recovery period of seven days, participants performed the Bruce treadmill protocol under two separate conditions. The first condition was unloaded, while the second involved wearing an external load in the form of a 10 kg loaded vest. Participants had a 48-hour rest period between the two tests conditions. In order to create significant physiological stress that could affect the exercise responses of elite athletes, a 10 kg vest was selected based on similar protocols found in the literature and preliminary tests conducted prior to the study. After the vest was put on, it was adjusted so that both shoulders were symmetrical and secured with belts to prevent slippage during the test. To minimize order effects, the conditions were administered in a counterbalanced sequence (Fig. 1). This approach ensured the reliability and validity of the results. This methodological approach facilitated the examination of load-dependent variations in physiological responses while controlling for potential order effects.

### Data collection tools

Height and body weight measurements were taken while participants stood barefoot and lightly clothed using a calibrated stadiometer and digital scale (Seca GmbH, Hamburg, Germany). Body mass index (BMI) was calculated using the standard formula: weight (kg)/height^2^ (m^2^). Body composition data (fat, fat mass, and fat-free body weight) were obtained using the BIA (bioelectrical impedance analysis) method with the InBody 770 (InBody, Seoul, South Korea) device. All measurements were performed in the morning, following an overnight fast, under standard conditions by the researchers.

### Isokinetic force measurement

Bilateral isokinetic muscle strength assessments were performed using a Biodex System 3 dynamometer (Biodex Medical Systems, Shirley, NY, USA), a highly validated device known for its exceptional reliability in torque measurement (ICC > 0.90). The testing protocol included evaluations at two angular velocities, each serving a distinct purpose: 60°/s to measure maximum force production capacity and 240°/s to assess muscular performance specific to power output. This dual-velocity approach provided a comprehensive profile of muscle functionality. This apparatus employs sophisticated servo-controlled mechanisms to maintain constant angular velocity throughout the range of motion, enabling precise quantification of torque output during both concentric and eccentric muscular contractions while controlling for velocity-dependent variations in force production^17^. The dynamometer was calibrated and configured to assess bilateral knee flexor and extensor muscle groups, with precise alignment of the anatomical axis of rotation with the mechanical axis of the device. Before testing, participants engaged in a standardized warm-up routine designed to reduce the risk of injury and enhance neuromuscular performance. The testing protocol featured two distinct assessments tailored to specific velocity parameters. Low-velocity testing at 60°/s was conducted to evaluate maximum force production capacity, while high-velocity testing at 240°/s targeted the assessment of power-endurance characteristics. This combination ensured a thorough analysis of muscular performance across varying demands. Standardized positioning and stabilization procedures were implemented, including adjustable restraints securing the tested limb and torso to minimize compensatory movements. The rotation axis was aligned with the lateral femoral epicondyle, and the resistance pad was positioned proximally to the lateral malleolus. Participants performed three maximal voluntary contractions at each angular velocity for both flexion and extension movements, with peak torque values being recorded for subsequent analysis. Standardized verbal encouragement was provided throughout the testing procedure to ensure maximal neuromuscular activation and performance consistency.

### Cardio-pulmonary endurance test

Cardiorespiratory parameters were quantified using a COSMED K5 metabolic analyzer (COSMED, Rome, Italy) during the implementation of the Bruce protocol, a standardized cardiopulmonary exercise testing methodology. The assessment protocol measured key physiological variables including maximal oxygen consumption (VO2max), average breath volume (ABV), average breath frequency (ABF), peak heart rate (HR-peak), and average oxygen density (AOD). Real-time gas exchange analysis was conducted using breath-by-breath technology via a fitted face mask (Hans Rudolph, Inc., Kansas City, USA). VO₂max was determined using a combination of standardized physiological criteria to ensure accurate assessment during the Bruce treadmill protocol. Specifically, VO₂max was considered achieved when participants met at least two of the following criteria: (i) a plateau in oxygen uptake despite increasing workload, defined as a change in VO₂ of less than 150 mL/min; (ii) a respiratory exchange ratio (RER) equal to or greater than 1.10; (iii) attainment of within ± 10 beats per minute of the age-predicted maximum heart rate (APHRmax = 220 − age); and (iv) volitional exhaustion, indicated by the participant’s decision to terminate the test despite verbal encouragement. Prior to testing, a comprehensive calibration procedure was executed according to manufacturer specifications, incorporating a 45-minute warm-up period for system stabilization. The calibration protocol included flowmeter verification using a 3 L volume-certified syringe and metabolic calibration with reference gases (5% CO2, balanced N2), with iterative volume calibrations performed until achieving less than 2% variance between consecutive measurements, ensuring optimal measurement accuracy and reliability^18,19^. Following a standardized pre-exercise protocol comprising a 10-minute functional warm-up and 5-minute dynamic stretching regimen, participants initiated the treadmill test at baseline parameters of 1.7 mph with a 10% grade inclination. The protocol implemented systematic increments at three-minute intervals, with grade elevation increasing by 2% and speed adjustments as detailed in Table 1. Continuous monitoring and recording of physiological parameters were maintained throughout each stage, including VO2max, ABV, ABF, HR-peak, and AOD. The termination criteria for the test during the Bruce protocol were determined in accordance with ACSM guidelines. These criteria include chest pain or anginal symptoms, dizziness, syncope, serious arrhythmias, excessive fatigue, and the participant’s request to terminate the test. Additionally, HR-peak measurements were taken and ECG monitoring was performed in our study; this allowed for the monitoring of potential cardiac risks, enabling the test to be safely terminated when necessary^20^.

**Table 1:** Bruce protocol.

Level	Speed (mph)	Incline (%)
1	1.7	10
2	2.5	12
3	3.4	14
4	4.2	16
5	5.0	18
6	5.5	20
7	6.0	22

### Statistical analyses

The statistical analyses were conducted using SPSS 26.0 software (SPSS Inc., Chicago, IL), ensuring robust examination of the data. For visual representation, GraphPad Prism 8.0.2 (GraphPad Software Inc., San Diego, CA, USA) was employed to create detailed and informative graphical designs. Results from the analyses were expressed in terms of mean values, standard deviations, percentage changes, mean differences, and effect sizes, providing a comprehensive overview of the findings. The effect size was calculated by Cohen’s d coefficient, and according to Cohen, a d value of less than 0.2 was considered as weak effect, around 0.5 as moderate effect, and greater than 0.8 as strong effect^21^. Accordingly, the normal distribution of the data was tested with the Shapiro-Wilk test. In addition, Q-Q plot graphs and boxplots were examined for visual analysis. Skewness and Kurtosis values were analyzed for parameters that did not show normal distribution (*p* < .05) in Shapiro-Wilk test results. Therefore, parametric tests were used in the study. Accordingly, the Paired Sample T Test was used to compare the cardiopulmonary test results obtained during the loaded and unloaded Bruce protocol exercise. A 95% confidence interval (CI) was given to determine the reliability of the results. Linear regression analysis was performed to determine the effect of isokinetic test results at angular velocities of 60°/s and 240°/s during loaded and unloaded exercise on ABV. The R^2^ coefficient was calculated to evaluate the fit of the model, and the significance of the model was tested with the F test. In addition, p-values were also recorded to understand the effect of each independent variable on the dependent variable (ABV). The significance level was set at 0.05.

## Results

**Table 2:** Demographic information of participants.

Parameters	M ± SD	Minimum	Maximum
Age (year)	21.43 ± 0.77	20.0	23.0
Height (cm)	178.06 ± 5.31	166.0	186.0
Weight (kg)	71.29 ± 7.24	57.6	91.5
BMI (kg/m^2^)	22.45 ± 2.11	18.6	26.4
Fat (%)	10.64 ± 3.85	2.7	18.0
Fat Mass (kg)	7.88 ± 3.4	1.6	14.7
FBW (kg)	63.41 ± 5.0	51.40	76.80

*BMI* body mass index, *FBW* fat-free body weight.

Table 2 shows the demographic characteristics of the participants. Accordingly, the mean age of the participants was 21.43 ± 0.77 years, mean height was 178.06 ± 5.31 cm, mean body weight was 71.29 ± 7.24 kg, mean BMI was 22.45 ± 2.11 kg/m^2^, mean FAT was 10.64 ± 3.85%, mean FAT MASS was 7.88 ± 3.4 kg, and mean FBW was 63.41 ± 5.0 kg.

**Table 3:** Comparison of VO2max and related parameters of participants according to loaded vs. unloaded aerobic capacity test.

Parameters	Loaded	Unloaded	t	Cohen’s d	*p*	95% CI	
						Lower	Upper
VO2max (ml/kg/min)	57.72 ± 3.90	59.52 ± 5.40	-1.869	-0.34	0.072	-0.70	0.03
ABV (L)	147.25 ± 20.67	149.32 ± 18.83	-0.607	-0.11	0.529	-0.46	0.24
VO_2_max (ml/kg/min)	60.15 ± 11.39	56.80 ± 9.77	1.939	0.35	0.062	-0.01	0.72
HR-peak (beats)	188.03 ± 7.71	192.20 ± 7.25	-3.551	-0.64	0.001	-1.03	-0.24
AOD (%)	16.92 ± 0.41	16.81 ± 0.44	1.667	0.30	0.106	-0.06	0.66

*ABV* average breath volume, *HR* heart rate, *AOD* average oxygen density, *VO2max* oxygen consumption

**Fig. 2:**
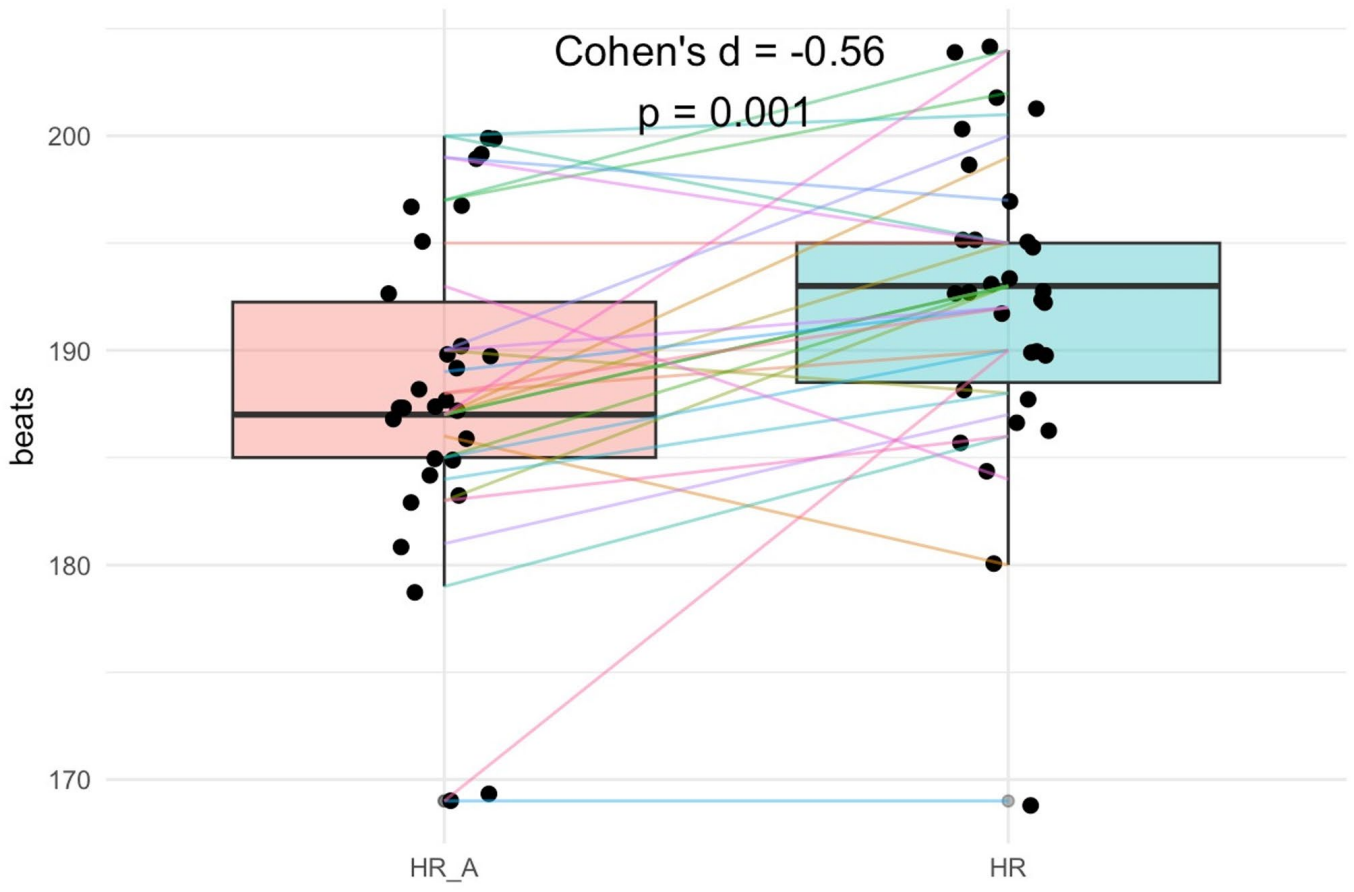
Comparison of participants’ heart rates during loaded and unloaded: HR_A = heart rate with loaded ACT, HR = heart rate in unloaded ACT.

Statistical analysis of cardiorespiratory parameters during loaded versus unloaded ACT, as presented in Table 3, revealed a significant elevation in heart rate response during aerobic capacity test [t = − 3.551, *p* = .001, Cohen’s d = − 0.64, 95% CI (− 1.03, − 0.24)] (Fig. 2). Although other physiological parameters, including VO2max, ABV, ABF and AOD, demonstrated small effect sizes, these variations did not reach statistical significance.

**Table 4:** Linear regression results between ABV during the loaded and unloaded ACT and 60-°/s knee joint isokinetic performance parameters.

Parameters	training type	B	Std. err.	Beta	t	*p*
PT-EXT-R	Loaded	2.883	0.804	3.522	3.522	0.003*
	Unloaded	1.567	1.102	2.137	1.421	0.176
PT-EXT-L	Loaded	− 1.786	0.771	− 3.303	− 2.317	0.035*
	Unloaded	− 0.920	1.056	− 1.868	− 0.871	0.397
PT-FLX-R	Loaded	− 3.566	1.178	− 4.300	− 3.028	0.008*
	Unloaded	− 1.003	1.614	− 1.328	− 0.622	0.543
PT-FLX-L	Loaded	3.234	1.144	4.185	2.826	0.013*
	Unloaded	1.038	1.568	1.474	0.662	0.518
TW-EXT-R	Loaded	− 0.006	0.076	− 0.044	− 0.085	0.933
	Unloaded	− 0.096	0.104	− 0.728	− 0.927	0.369
TW-EXT-L	Loaded	− 0.116	0.091	− 0.962	− 1.272	0.233
	Unloaded	− 0.105	0.125	− 0.953	− 0.838	0.415
TW-FLX-R	Loaded	0.001	0.110	0.008	0.012	0.990
	Unloaded	− 0.012	0.151	− 0.074	− 0.077	0.940
TW-FLX-L	Loaded	0.285	0.112	1.666	2.545	0.022*
	Unloaded	0.256	0.154	1.640	1.666	0.116
AP-EXT-R	Loaded	− 0.171	0.471	− 0.177	− 0.364	0.721
	Unloaded	0.129	0.645	0.146	0.200	0.844
AP-EXT-L	Loaded	1.366	0.682	1.681	2.004	0.063
	Unloaded	1.015	0.934	1.371	1.087	0.294
AP-FLX-R	Loaded	0.394	0.681	0.306	0.578	0.572
	Unloaded	0.665	0.933	0.568	0.715	0.487
AP-FLX-L	Loaded	− 3.426	1.036	− 2.720	− 3.307	0.005*
	Unloaded	− 2.754	1.419	− 2.400	− 1.941	0.071
AGANT-R	Loaded	7.919	2.597	4.600	3.050	0.008*
	Unloaded	3.255	3.557	2.075	0.915	0.375
AGANT-L	Loaded	− 4.406	2.290	− 2.683	− 1.924	0.074
	Unloaded	− 1.770	3.137	− 1.173	− 0.564	0.581

*PT* peak tork, *TW* total work, *AP* average power, *AGANT* agonist muscle, *EXT* extension, *FLX* flexion, *R* right, *L* left.

In Table 4, the results of linear regression between ABV and 60-degree knee joint isokinetic performance parameters of the participants during loaded and unloaded ACT exercises were analyzed. According to this, 60 degrees of isokinetic knee joint movements significantly explained the loaded ABV results (R^2^ = 804, *p* = .004). According to these results, PT-EXT-R (t = 3.522, *p* = .003), PT-EXT-L (t=− 2.317, *p* = .035), PT-FLX-R (t=− 3.028, *p* = .008), PT-FLC-L (2.826, *p* = .013), TW-FLX-L (t = 2.545, p=. 022), AP-FLX-L (t = 3.307, *p* = .005), AGANT-R (t = 3.050, *p* = .008) significantly explained the ABV results during loaded ACT exercise. However, there was no relationship between ABV and any isokinetic strength parameter during unloaded ACT.

**Table 5:** Linear regression results between ABV during the loaded and unloaded ACT and 240-°/s knee joint isokinetic performance parameters.

Parameters	Training type	B	Std. err.	Beta	t	*p*
PT-EXT-R	Loaded	− 0.028	1.249	− 0.022	− 0.023	0.982
	Unloaded	-1.203	1.775	-1.030	− 0.678	0.508
.PT-EXT-L	Loaded	1.660	1.122	1.413	1.480	0.160
	Unloaded	− 0.760	1.595	− 0.710	− 0.476	0.641
PT-FLX-R	Loaded	− 0.192	1.297	− 0.152	− 0.148	0.884
	Unloaded	0.338	1.843	0.292	0.183	0.857
PT-FLX-L	Loaded	− 0.393	1.406	− 0.284	− 0.280	0.783
	Unloaded	2.833	1.999	2.237	1.412	0.178
TW-EXT-R	Loaded	0.228	0.068	2.857	3.333	0.005*
	Unloaded	− 0.033	0.097	− 0.452	− 0.338	0.740
TW-EXT-L	Loaded	− 0.087	0.046	-1.337	-1.892	0.078
	Unloaded	− 0.019	0.066	− 0.316	− 0.287	0.778
TW-FLX-R	Loaded	− 0.166	0.057	-2.344	-2.886	0.011*
	Unloaded	− 0.010	0.082	− 0.151	− 0.119	0.907
TW-FLX-L	Loaded	0.079	0.035	1.148	2.265	0.039*
	Unloaded	0.047	0.050	0.747	0.945	0.360
AP-EXT-R	Loaded	-2.747	0.845	-3.592	-3.251	0.005*
	Unloaded	0.160	1.201	0.230	0.133	0.896
AP-EXT-L	Loaded	1.124	0.552	1.624	2.035	0.060
	Unloaded	0.150	0.785	0.238	0.191	0.851
AP-FLX-R	Loaded	2.098	0.645	3.228	3.255	0.005*
	Unloaded	0.467	0.916	0.788	0.509	0.618
AP-FLX-L	Loaded	-1.706	0.480	-2.461	-3.557	0.003*
	Unloaded	− 0.889	0.682	-1.408	-1.304	0.212
AGANT-R	Loaded	0.072	1.496	0.052	0.048	0.962
	Unloaded	-1.372	2.126	-1.086	− 0.646	0.528
AGANT-L	Loaded	1.780	1.526	1.167	1.166	0.262
	Unloaded	-1.837	2.169	-1.322	− 0.847	0.410

*PT* peak tork, *TW* total work, *AP* average power, *AGANT* agonist muscle, *EXT* extension, *FLX* flexion, *R* right, *L* left.

In Table 5, the results of linear regression between ABV during the loaded and unloaded Bruce Protocol and 240 degrees/sec knee and joint isokinetic performance parameters were analyzed. Accordingly, 240 degrees/sec isokinetic knee joint movements significantly explained the loaded ABV results (R2 = 0.761, *p* = .012). According to these results, in the loaded ACT training group, TW-EXT-R (t = 3.333, *p* = .005), TW-FLX-R (t=− 2.886, *p* = .011), TW-FLX-L (t = 2.265, p=. 039), AP-EXT-R (t=-3.251, *p* = .005), AP-FLX-R (t = 3.255, *p* = .005), AP-FLX-L (t=− 3.557, *p* = .003) significantly explained ABV. However, no correlation was found between ABV during the unloaded ACT and other isokinetic strength parameters.

## Discussion

The primary objective of this study was to investigate the relationship between isokinetic knee muscle strength measured at angular velocities of 60°/s and 240°/s and cardiovascular parameters (e.g., tidal volume, respiratory rate, and oxygen consumption) recorded during aerobic capacity test (ACT) performed both without weights and with a 10 kg vest. Our research hypothesis was that bilateral isokinetic knee strength could significantly predict cardiorespiratory responses, particularly during the ACT protocol with a weight vest, whereas this relationship would be more limited in the unloaded protocol. The findings partially support this hypothesis: Statistically significant correlations were found between knee strength and certain cardiorespiratory variables during the loaded ACT application. However, in the protocol using body weight, these relationships either remained at lower levels or did not reach statistical significance. For instance, comparative analyses of moderate-intensity continuous exercise and both low- and high-volume exercise protocols have revealed significantly elevated heart rate responses during ACT sessions, underscoring its acute physiological and psychological benefits^22^ Furthermore, while high-intensity resistance exercises have their merits, they tend to be less effective in improving key cardiorespiratory metrics—such as maximal oxygen consumption (VO2max) and mean heart rate—when compared to ACT protocols^23^. Notably, ACT has been shown to enhance cardiac autonomic modulation by improving the balance between sympathetic and parasympathetic activity. This, in turn, promotes better heart rate variability (HRV), a well-established marker of cardiovascular health and endurance capacity^24^. Although the physiological mechanisms that underpin the superior effects of ACT on cardiac parameters have been extensively studied, a critical gap persists in understanding how isokinetic muscular strength—specifically at angular velocities of 60°/s and 240°/s—correlates with cardiorespiratory responses during loaded and unloaded ACT protocols. To our knowledge, this study is the first to investigate these specific associations, offering new insights into the interplay between muscular strength and cardiorespiratory performance during ACT. By addressing this gap, the findings have the potential to inform more nuanced exercise prescriptions and training strategies for optimizing both muscular and cardiovascular outcomes.

Analysis of cardiorespiratory parameters during loaded and unloaded running-based ACT revealed a significant difference in HR response favoring unloaded exercises, while no significant differences were observed in VO2max, ABV, ABF, and AOD. These findings demonstrate that unloaded ACT protocols elicit higher heart rate responses compared to loaded protocols. The current literature presents diverse evidence regarding the impact of ACT interventions on cardiorespiratory parameters, contributing to the ongoing discourse in this field. The study examining the acute effects of a ACT protocol including resistance exercises and a traditional ACT protocol performed on a bicycle ergometer or treadmill on cardiac biomarkers^24^ and another study examining the effects on physiological and perceptual responses^25^ reported no significant difference in HR values between the two intervention groups.

Significant methodological variations exist across these investigations, particularly in resistance exercise protocols (e.g., squat exercises calibrated to 20% of body weight and cycle ergometry interventions). The heterogeneity of ACT protocols is particularly significant, as different modalities of high-intensity interval training can lead to distinct cardiorespiratory adaptations and outcomes^26^. Empirical evidence underscores the importance of factors such as intensity, duration, and modality in shaping the physiological responses and overall effectiveness of ACT programs^27^. Notably, functional ACT protocols that incorporate whole-body aerobic exercises—such as planks, squats, push-ups, and sit-ups—have been shown to impose substantial oxygen consumption (VO2) demands. This elevated VO2 requirement, in turn, plays a pivotal role in driving mean heart rate responses during these sessions. This physiological relationship underscores the metabolic demands associated with multi-joint, compound movements within high-intensity training paradigms^28^. The variability in cardiorespiratory parameters may be attributed to these methodological factors, highlighting the protocol-dependent nature of physiological responses in exercise interventions.

The present investigation focuses on the potential metabolic effects of running-based ACT protocols, both loaded and unloaded. Traditional running-based ACT exercises demonstrate elevated heart rate responses compared to other ACT modalities, primarily attributed to higher ratings of perceived exertion (RPE). This physiological distinction underscores the unique cardiovascular demands associated with running-based protocols within the ACT paradigm^28^. In other words, the increased difficulty level may have resulted in higher HR. This may explain the increase in HR value of running-based ACT exercises. However, when comparing loaded and unloaded ACT exercises, the general opinion is that loaded ACT exercises affect cardiorespiratory parameters^29^ by increasing heart rate due to the additional load on the musculoskeletal system^30,31^.

The incorporation of external loads during walking can augment metabolic costs and relative exercise intensity; however, the relationship between metabolic demand and increases in speed and load is non-linear. To optimize metabolic cost elevation, individuals employing slower walking velocities require greater incremental load progressions, whereas those maintaining faster walking speeds necessitate smaller load increments. This inverse relationship between walking velocity and load progression demonstrates the complex interplay of biomechanical and metabolic factors in loaded ambulatory exercise^29^. These findings suggest that the external load utilized during loaded versus unloaded ACT protocols may significantly influence metabolic outcomes. Consequently, the specific loads implemented in this investigation may have attenuated the achievement of maximal heart rate responses, indicating a potential load-dependent modulation of cardiovascular strain during high-intensity interval training interventions.

In accordance with the present investigation, no significant differences were observed in VO2max, PBV, PBF and AOD parameters. This absence of significant variation may be attributed to the running-based nature of both ACT protocols implemented throughout the study period. Such locomotor activities necessitate greater muscular power output and increased range of motion in lower extremity muscle groups, characteristics inherent to running-based exercises regardless of protocol variations^32^. Exercises predominantly engaging lower extremity musculature elicit significantly higher oxygen consumption (VO_2_) requirements compared to those primarily utilizing upper extremity muscle groups, reflecting the greater metabolic demands associated with larger muscle mass recruitment and activation patterns^23^. Running-based ACT protocols elicit substantially elevated oxygen consumption (VO2) levels compared to bodyweight-based ACT interventions, demonstrating the enhanced metabolic demands inherent to running-specific locomotor patterns^33^. This evidence shows that the muscle groups active during exercise are decisive on VO_2_^34,35^. The fact that both protocols were running-based may have suppressed the possible effect of the loaded ACT protocols.

A significant finding of this investigation reveals a correlation between knee isokinetic muscle strength at angular velocities of 60°/s and 240°/s and average breath volume (ABV) measured during loaded and unloaded ACT exercises. Isokinetic muscle strength at both angular velocities demonstrates explanatory power for ABV values obtained during ACT protocols. At 60°/s angular velocity, knee isokinetic strength performance correlates with ABV values measured during loaded ACT performance across multiple parameters, including PT-EXT-R, PT-EXT-L, PT-FLX-R, PT-FLC-L, TW-FLX-L, AP-FLX-L, and AGANT-R. Conversely, at 240°/s angular velocity, significant correlations were identified with TW-EXT-R, TW-FLX-R, TW-FLX-L, AP-EXT-R, AP-FLX-R, and AP-FLX-L parameters. The findings suggest that knee isokinetic muscle strength performance at angular velocities of 60°/s and 240°/s significantly influences respiratory functions during loaded ACT exercises, yielding superior outcomes compared to unloaded ACT protocols. Supporting this conclusion, Marzorati et al. (2000) demonstrated that knee isokinetic exercises performed at 60°/s and 180°/s angular velocities enhanced respiratory functions in healthy individuals. Interestingly, exercises conducted at 60°/s angular velocity produced greater power output compared to those performed at 180°/s, highlighting the importance of angular velocity in optimizing performance outcomes^36^. In a related study, Akinoglu et al. explored the intricate relationship between peripheral muscle strength, respiratory muscle strength, and pulmonary function in elite athletes from various sports disciplines. Their investigation assessed isokinetic muscle strength at angular velocities of 60°/s and 180°/s, focusing on peak torque values derived from PBV parameters. These findings collectively underscore the critical role of isokinetic muscle strength in modulating respiratory and pulmonary functions across different exercise modalities and populations. Their findings demonstrated a concurrent development pattern between knee isokinetic muscle strength, respiratory functions, and respiratory musculature, indicating synchronized adaptations across these physiological systems^37^. Liu et al. approached the relationship between isokinetic muscle contractions and cardiorespiratory functions from a novel angle, focusing specifically on upper extremity isokinetic muscle strength at angular velocities of 60°/s and 180°/s. Their study revealed significant effects of upper extremity strength on respiratory and pulmonary functions, identifying distinct correlations: elbow flexor strength was strongly associated with inspiratory muscle power, while elbow extensor strength showed a notable relationship with expiratory muscle power^38^. These findings highlight the integral role of upper extremity isokinetic strength in influencing respiratory mechanics, offering a fresh perspective on the interplay between muscular and cardiorespiratory systems. While aligning with existing evidence, the present investigation extends these findings considerably. Whereas previous studies primarily focused on the relationship between isokinetic muscle strength and cardiorespiratory fitness across diverse populations, this investigation specifically examined the association between isokinetic muscle strength and ABV parameters during loaded and unloaded ACT exercises. Consistent with the literature, isokinetic muscle strength at 60°/s angular velocity demonstrated improvements in ABV parameters. Additionally, this study revealed that isokinetic muscle strength at 240°/s angular velocity significantly influenced ABV parameters during loaded ACT exercises, contributing novel insights to the existing body of knowledge.

The significant relationship between isokinetic muscle strength around the knee and ABV during the ACT protocol can be explained by multiple physiological mechanisms. First, stronger lower extremity muscles can provide more efficient ventilation during increased exercise intensity, thereby reducing the workload of respiratory muscles and improving ventilatory efficiency^39^. This effect becomes more pronounced in load-dependent protocols in response to increased metabolic demands. In addition, increased mechanical and metabolic stress during loaded ACT requires greater motor unit activation and rapid muscle fiber (type II) recruitment. This enables more effective use of both lower extremity muscles and accessory respiratory muscles, which may contribute to increased ABV^40^. From another physiological perspective, ACT creates significant metabolic stress, increasing the production of metabolites such as lactate and hydrogen ions. Individuals with greater muscle strength better buffer these metabolites, maintaining acid-base balance and delaying fatigue. This capacity enhances oxygen utilization efficiency, reduces the need for excessive ventilation, and supports higher ABV^41^. During intense exercise, peripheral muscle fatigue can increase the urge to breathe through afferent feedback mechanisms. Stronger muscles feel less fatigue, which reduces this feedback and prevents unnecessary increases in ventilation. This moderation becomes more stable during loaded ACT and contributes to high ABV^42^.

Several limitations warrant consideration in the present investigation. Primarily, the study cohort comprised a relatively small and homogeneous population (mean age: 21.43 ± 0.77 years), potentially limiting the generalizability of findings to populations with broader age ranges and fitness levels. Furthermore, the exclusive inclusion of healthy individuals may restrict the validity of results for populations with sports-related injuries or knee pathologies. Additionally, while the ACT protocols were standardized for duration and intensity, individual physiological variations (e.g., muscle fiber composition, metabolic rate) may have influenced the outcomes. Future investigations should examine these relationships across more diverse sample populations and varying ACT durations to establish broader clinical applicability. Another limitation of our study is that the weight vests used in the load test phase were not customized for each participant. Future studies could use weights indexed according to a percentage of the participant’s 1RM and body composition.

## Conclusions

This investigation examined the effects of knee isokinetic strength performance on cardiorespiratory parameters during loaded and unloaded ACT protocols. The findings demonstrate that knee isokinetic strength performance at angular velocities of 60°/s and 240°/s significantly explains cardiorespiratory parameters measured during loaded ACT. Notably, no such correlations were identified in unloaded ACT protocols, and heart rate (HR) measurements were consistently lower during unloaded ACT sessions.

## Data Availability

The datasets generated and/or analyzed during the current research are available from the corresponding author on reasonable request.
